# Biocontrol of *Phytophthora* Root and Stem Rot and Growth Promotion of Soybean Plants by the Rhizobacterium *Enterobacter pseudoroggenkampii* Strain GVv1 Isolated from *Vicia villosa* Roth

**DOI:** 10.1264/jsme2.ME24089

**Published:** 2025-05-01

**Authors:** Juan Taboadela-Hernanz, Yuichiro Ikagawa, Kosei Yamauchi, Yui Minoshima, Haruhisa Suga, Masafumi Shimizu

**Affiliations:** 1 Laboratory of Plant Pathology, The United Graduate School of Agricultural Science, Gifu University, 1–1 Yanagido, Gifu-shi, Gifu 501–1193, Japan; 2 Food Microbiology Laboratory, The United Graduate School of Agricultural Science, Gifu University, 1–1 Yanagido, Gifu-shi, Gifu 501–1193, Japan; 3 Natural Products Chemistry Laboratory, Faculty of Applied Biological Sciences, Gifu University, 1–1 Yanagido, Gifu-shi, Gifu, 501–1193, Japan; 4 Laboratory of Plant Pathology, Faculty of Applied Biological Sciences, Gifu University, 1–1 Yanagido, Gifu-shi, Gifu 501–1193, Japan; 5 Institute for Glyco-core Research (iGCORE), Gifu University, 1–1 Yanagido, Gifu-shi, Gifu 501–1193, Japan

**Keywords:** biocontrol, *Phytophthora sojae*, soybean, daidzein, *Enterobacter*

## Abstract

*Phytophthora* root and stem rot (PRSR) caused by *Phytophthora sojae* is a major concern for global soybean production. To identify a bacterial biocontrol agent against PRSR, 73 rhizobacterial strains were isolated from wild and cultivated legumes and screened for their protective activities against PRSR in pot experiments. Strain GVv1 was selected for its consistent protective effect through repeated pot experiments. The protective effect of this strain was similar to that of the fungicide mancozeb-metalaxyl. A dual-culture assay showed that GVv1 produced antifungal metabolites effective against *P. sojae*. To evaluate the potential adaptability of GVv1 to the soybean rhizosphere environment, its growth was exami­ned in soybean root exudates and nutrient medium, both supplemented with daidzein, an antimicrobial isoflavone secreted by soybean roots. GVv1 proliferated using soybean root exudates and had sufficient tolerance to daidzein to colonize the soybean rhizosphere. The plant growth-promoting effect of GVv1 on soybean plants was also investigated. GVv1 significantly increased shoot and root dry weights, indicating its plant growth-promoting activity. *In vitro* assays showed that GVv1 produced indole-3-acetic acid, siderophores, and 1-aminocyclopropane-1-carboxylate deaminase and solubilized insoluble phosphates. A taxonogenomic ana­lysis of the draft genome identified GVv1 as *Enterobacter pseudoroggenkampii* with high similarity (98.32% average nucleotide identity) to *E. pseudoroggenkampii* strain 155092^T^. To the best of our knowledge, this is the first study to report the biocontrol and plant growth-promoting activities of *E. pseudoroggenkampii*.

*Phytophthora* root and stem rot (PRSR) of soybean (*Glycine max*), caused by *Phytophthora sojae* Kauffmann and Gerdemann, is one of the most devastating soil-borne diseases affecting soybean production, particularly in poorly drained fields (Dorrance, 2018). PRSR has been prevalent for >70 years since it was first reported in Indiana (United States) in 1948 (Schmitthenner, 1985) and has consequently been reported in many production areas worldwide (McCoy *et al.*, 2023). PRSR is responsible for enormous losses, estimated at USD 1 to 2 billion annually, with a global average annual loss of >1.1 million metric tons (Tyler, 2007; Allen *et al.*, 2017; Dorrance, 2018).

PRSR typically occurs in early spring, coinciding with the soybean seedling stage, which is more susceptible to the disease; however, it may occur at any development stage (Dorrance *et al.*, 2007; Akamatsu *et al.*, 2019). In the early stages, *P. sojae* infection manifests as seed rot or seedling damping-off. During the late season, infected soybean plants begin to show brown to dark stem canker from the roots up to the stem, combined with stunted growth, leaf yellowing, and premature plant death (Dorrance, 2018).

*P. sojae*, similar to other oomycete plant pathogens, requires a damp environment and saturated soil conditions for zoospore development and dispersal towards the host plant (Gijzen and Qutob, 2009). Under these conditions, disease severity may be markedly exacerbated, potentially leading to total yield loss. Consequently, improving soil drainage and compaction and avoiding planting before anticipated heavy storms that may lead to flooding or saturated soil conditions have been recommended as effective management strategies (Dorrance, 2018). However, the ability of *P. sojae* to survive in the soil for long periods in the form of resistant structures reduces the effectiveness of these practices (Dorrance *et al.*, 2008). The integration of resistant cultivars, major resistance (*Rps*: resistance to *P. sojae*) genes, and QTL-mediated resistance have also contributed to combating the disease (Sugimoto *et al.*, 2012). Nevertheless, this is insufficient to achieve complete protection against PRSR. A recent survey by McCoy *et al.* (2023) between 1990 and 2019 in the United States, Argentina, Canada, and China reported a decrease in the efficacy of specific *Rps* genes and a significant increase in pathotype diversity. The most effective practice widely used to control PRSR is seed treatment with fungicides, such as metalaxyl, mefenoxam, ethaboxam, and oxathiapiprolin (Dorrance and McClure, 2001). However, the increasing emergence of *P. sojae* with resistance to these fungicides (Peng *et al.*, 2019; McCoy *et al.*, 2022) and public concerns about the use of chemical pesticides have fueled efforts to reduce fungicide use and develop environmentally friendly alternatives. Biocontrol using antagonistic microorganisms is increasingly being implemented as an alternative that may contribute to establishing an integrated pest management strategy that reduces reliance on chemical control.

Various strains of different microbial taxa exhibit antagonistic activity against *P. sojae* under *in vitro* conditions; however, only a few, such as *Bacillus altitudinis* JSCX-1 and *Streptomyces* sp. S11, exhibit biocontrol activity against PRSR on soybean plants (Lu *et al.*, 2017; Arfaoui *et al.*, 2018). It is important to note that none of these antagonistic microorganisms are commercially available.

The present study was conducted to identify a novel biocontrol agent (BCA) against PRSR of soybean plants. To achieve this, bacteria from the rhizosphere soil of cultivated and wild legume plants were isolated and screened for their PRSR control activity.

## Materials and Methods

### Isolation of rhizobacteria from legume plants

Seven species of wild and cultivated legumes, as listed in Table 1, were collected from different sites in 2022 and 2023. Soil particles loosely attached to the roots were removed, and the remaining soil firmly attached to the roots was considered to be rhizosphere soil. Ten grams of root samples with rhizosphere soil were suspended in 100‍ ‍mL of sterilized distilled water (SDW), shaken for 30‍ ‍min, and filtered through filter paper (qualitative filter paper No. 1; Advantec). Since soybean secretes the antimicrobial isoflavone, daidzein, from its roots (Okutani *et al.*, 2020), daidzein-tolerant or daidzein-utilizing bacteria may be suitable as BCAs. Therefore, in the present study, rhizobacteria that grow in medium supplemented with daidzein were isolated from each plant sample as follows: 100‍ ‍μL of the above rhizosphere soil suspension was added to 3‍ ‍mL of 1/4 strength M63‍ ‍minimal medium (Pardee *et al.*, 1959) containing 20‍ ‍μM daidzein and 0.6‍ ‍mM cycloheximide (antifungal antibiotic) and incubated at 30°C for 72‍ ‍h with shaking at 200‍ ‍rpm. The culture broth was serially diluted, and a 100-μL aliquot of each dilution was spread on a 9-cm plate of tryptic soy agar (TSA) medium (Difco) and incubated at 30°C for 24 h. Bacterial colonies on the plates were purified by the quadrant streaking method, and a pure culture of each bacterial strain was stored in 20% glycerol at –‍80°C until used.

### Inoculum preparation of the pathogen and rhizobacteria

In the present study, *P. sojae* isolate TYMJ2, isolated from diseased soybean plants grown in the field naturally infested with *P. sojae* in Toyama Prefecture, Japan, was used as the challenge patho­gen. The zoospore suspension and oospore inoculum of TYMJ2 were prepared for experiments.

The zoospore suspension was prepared as follows. Field soil was suspended in a 100-fold volume of tap water, shaken for 10‍ ‍min, filtered through filter paper (qualitative filter paper No. 2; Advantec), and autoclaved twice (at 101°C for 15‍ ‍min and then at 120°C for 20‍ ‍min). This soil extract was diluted 5-fold with SDW and used as the incubation solution. TYMJ2 was grown on soybean extract agar (SEA) medium (Matsuoka *et al.*, 2021) at 25°C for 7 days. Eighteen mycelial agar plugs (diameter of 5‍ ‍mm) were cut from the edge of the colony and transferred to a 9-cm glass Petri dish containing 60‍ ‍mL of the above incubation solution. The dish was incubated at 21.5°C for 8‍ ‍h under the light, and the incubation solution containing zoospores and mycelia was filtered through two layers of gauze to remove mycelia. The concentration of the resulting zoospore suspension ranged between ~100 and 200 zoospores mL^–1^.

The oospore inoculum was prepared as follows. Eight mycelial agar plugs (diameter of 5‍ ‍mm) cut from the edge of a 7-day-old colony of TYMJ2 on SEA medium were inoculated into an autoclaved wheat bran medium (wheat bran, 60 g; 0.1% [w/v] sucrose solution, 100‍ ‍mL) in a 500-mL Erlenmeyer flask and incubated at 25°C for 21 days.

The stock solution of each bacterial strain was plated on TSA medium and incubated at 30°C for 24 h. A loopful of bacterial cells was transferred to Luria-Bertani (LB) liquid medium (Kanto Chemical) and incubated with shaking at 200‍ ‍rpm at 30°C for another 24 h. Bacterial cells were harvested by centrifugation (6,574×*g*, 10‍ ‍min), resuspended into a 10‍ ‍mM MgCl_2_·6H_2_O solution, and the concentration was adjusted by measuring the optical density at 600‍ ‍nm (OD_600_).

### Primary screening of candidate biocontrol strains

In primary screening, the biocontrol activities of rhizobacterial strains against PRSR were evaluated in a pot experiment under controlled environmental conditions. The soybean cultivar Enrei, susceptible to *P. sojae*, was used in the present study. Seeds were sown in 42-cell trays (Beepot Y-49; Canelon Kakou) filled with a double-autoclaved mixture of commercial soil (Saika-ichiban, Ibigawa Kogyo) and vermiculite at a 1:1 (v/v) ratio. A 4-mL aliquot of the cell suspension (OD_600_=1.0) of each rhizobacterial strain was applied to the soil mixture around the soybean seed in each cell. In a control treatment, an equal volume of 10‍ ‍mM MgCl_2_·6H_2_O was applied. Seeds were germinated at 27°C for 4 days under a 16-h light/8-h dark cycle in a controlled environment chamber (Biotron LH-200; Nippon Medical and Chemical Instruments). Seedlings were transplanted to 150-mL pots containing 100‍ ‍mL of a double-autoclaved commercial soil-vermiculite mixture and drench inoculated with a 10-mL zoospore suspension of TYMJ2. To facilitate pathogen infection, these pots were flooded with tap water to ~1‍ ‍cm above the soil surface every 3 days from the day of transplanting. Inoculated seedlings were grown in a controlled environment chamber (23°C, 16-h light/8-h dark cycle) for 5 days. The disease severity of the seedlings was scored based on root discoloration on a scale of 0 to 4 (0: healthy or no apparent discoloration, 1: <25% discoloration of the root, 2: 25% to 50% discoloration of the root, 3: 50% to 75% discoloration of the root, and 4: >75% discoloration of the root [Xi *et al.*, 2022]). The control efficacy of each bacterial treatment was calculated based on the following formula: Control efficiency=(1−[Mean disease score of the bacterial treatment]/[Mean disease score of the control treatment])×100. Each treatment had four replicate pots.

In this screening step, the pot experiment was repeated twice. Trial 1 tested the biocontrol efficacy of all bacterial strains, while trial 2 tested the biocontrol efficacy of the strains that showed >50% control efficiency in trial 1.

### Second screening of candidate biocontrol strains

Bacterial strains that consistently exhibited >50% control efficiency across the two trials in the above primary screening trials were subjected to a second screen conducted under greenhouse conditions between September and October 2023. In this screening, soybean seedlings were inoculated with the oospore inoculum to simulate *P. sojae* natural infection.

Soybean seeds (cv. Enrei) were sown in 42-cell trays filled with a non-autoclaved commercial soil-vermiculite mixture. Seeds were treated with the selected bacterial strain or MgCl_2_·6H_2_O solution and incubated in the controlled environment chamber, as described above. After 4 days of sowing, seedlings were transplanted to 150-mL pots containing 100‍ ‍mL of field soil inoculated with ~8.2% (w/w) of the TYMJ2 oospore inoculum. To facilitate pathogen infection, these pots were flooded with tap water to ~1‍ ‍cm above the soil surface every 3 days from the day of transplanting. The average temperature and relative humidity inside the greenhouse during the experimental period were 20.4°C and 63.5%, respectively. The control efficiency of each bacterial treatment was calculated at 9 days post-inoculation (dpi), as in the primary screening. Five replicate pots were prepared for each treatment. The experiment was repeated twice.

### Comparison of the disease control effect of strain GVv1 to the fungicide mancozeb-metalaxyl

In this experiment, the disease control efficacy of GVv1 was compared to that of the chemical fungicide mancozeb-metalaxyl, which is commonly used to manage PRSR. The experiment was conducted between May and June 2024.

Soybean seeds (cv. Enrei) were sown in 150-mL pots containing a 130-mL mixture of field soil and vermiculite at a 1:1 (v/v) ratio. Each seed was treated with a 4-mL cell suspension (OD_600_=1.0) of GVv1. As the fungicide treatment, 4‍ ‍mL of 1,000-fold diluted mancozeb-metalaxyl (Ridomil Gold MZ WG; Syngenta) was applied to the soil mixture surrounding the seed. In a control treatment, an equal volume of 10‍ ‍mM MgCl_2_·6H_2_O solution was applied. After 4 days of cultivation in the greenhouse, seedlings were challenged with 10‍ ‍mL of the *P. sojae* zoospore suspension and flooded with tap water to ~1‍ ‍cm above the soil surface every 3 days from the day of the pathogen inoculation. The average temperature and humidity inside the greenhouse during the experimental period were 22.3°C and 65.6%, respectively. Control efficiency was calculated at 9 dpi, as described above. Each treatment had 8 replicate pots. The experiment was repeated twice (referred to as trials 1 and 2). The difference in disease severity among the treatments was analyzed by the Mann–Whitney U test (*P*<0.05).

### Hemolytic activity

To investigate the potential risk of GVv1 against human and animal health, its hemolytic activity was assessed by a sheep blood agar test (Thakkar *et al.*, 2015). A loopful of GVv1 cells was streaked onto the surface of 5% sheep blood agar medium (Nippon Becton Dickinson) and incubated at 37°C for 48 h. The plates were visually inspected for a clear zone or color change around the colony, as described by Amaria *et al.* (2023).

### Inhibitory activity of GVv1 against *P. sojae* mycelial growth

A dual-culture assay was performed to investigate the inhibitory activity of GVv1 against *P. sojae* mycelial growth. GVv1 was streak inoculated 2‍ ‍cm from the edge of a 9-cm plate of potato dextrose agar (PDA). An agar plug (diameter of 5‍ ‍mm) cut with a cork borer from actively growing mycelia of TYMJ2 on the SEA plate was placed 2‍ ‍cm from the edge of the opposite side of the PDA plate. The control plate was inoculated with TYMJ2 only. The inhibitory activities of the less or non-protective strains GVh3, GVh5, and GVs5, which exhibited no or weak (<15%) control efficiency in primary screening, were also exami­ned for comparison. Plates were incubated at 25°C for 12 days. There were three replicate plates per treatment. The radius of the TYMJ2 colony in the direction of the bacterial inoculation site was measured. Data were analyzed for significant differences using Tukey’s HSD test (*P*<0.05).

### Ability of GVv1 to grow on soybean root exudates and nutrient medium supplemented with daidzein

This experiment aimed to investigate the potential adaptability of GVv1 to the soybean rhizosphere by monitoring GVv1 growth with the exogenous application of daidzein on nutrient medium and soybean root exudates.

Soybean root exudates were collected as follows: soybean seeds (cv. Enrei) were germinated in 42-cell trays filled with a double-autoclaved mixture of commercial soil and vermiculite at a 1:1 (v/v) ratio and grown at 27°C for 4 days under a 16-h light/8-h dark cycle in a controlled environmental chamber (Biotron LH-220S; Nippon Medical and Chemical Instruments). Soybean seedlings were uprooted, carefully washed with SDW to remove adherent soil particles, and transferred to a 250-mL Erlenmeyer flask containing 150‍ ‍mL SDW. After a 48-h incubation under the same conditions, the root exudates were collected, filter-sterilized with a 0.22-μm membrane filter (AS ONE), and stored at 4°C for later use.

The GVv1 cell suspension was inoculated into 10-fold diluted LB broth medium and soybean root exudates at a final concentration of OD_630_=0.07. Then, 10‍ ‍mM daidzein (Wako Pure Chemical Industries) prepared in dimethyl sulfoxide (DMSO) was added to GVv1-inoculated LB broth and root exudates to a final concentration of 10‍ ‍μM. As the control treatment, an equal volume of DMSO was added instead of daidzein. These broth media and root exudates were added in triplicate to a 96-well plate at 100‍ ‍μL well^–1^ and incubated at 30°C with shaking at 200‍ ‍rpm. OD_630_ was measured after a 48-h incubation. The growth of less or non-protective strains (*i.e.*, GVh3, GVh5, and GVs5) was also exami­ned for comparison. Differences in the OD_630_ value between treatments were analyzed using a one-way ana­lysis of variance and Tukey’s HSD test (*P*<0.05).

### Plant growth-promoting activity of GVv1 on soybean

Soybean seeds (cv. Enrei) were sown in 9-cm plastic pots filled with a mixture of commercial and field soil at a ratio of 1:1 (v/v). An 8-mL aliquot of the cell suspension (OD_600_=1.0) of GVv1 was drenched into the soil mixture of each pot around the soybean seed. In a control treatment, an equal volume of 10‍ ‍mM MgCl_2_·6H_2_O solution was drenched. These plants were grown in the greenhouse for 24 days after the inoculation. The average temperature and humidity inside the greenhouse during the growth period were 22.9°C and 60.7%, respectively. Soybean plants were uprooted from the pots, and their roots were gently washed with running tap water to remove soil particles. After removing excess water by leaving plants on filter paper for 15‍ ‍min, the cotyledons were removed from the plants because some plants had already defoliated the cotyledons, which may have affected the results obtained. Root and shoot samples were dried at 60°C for 24‍ ‍h in a natural convection dryer (NDO-420; EYELA) and weighed. Differences in root and shoot dry weights between the control and GVv1 treatments were analyzed by the Student’s *t*-test (*P*<0.05).

### Characteristics of GVv1 related to plant growth-promoting activity

#### Assessment of indole-3-acetic acid (IAA) production

The ability of GVv1 to produce IAA was exami­ned as described by Kumar *et al.* (2012) with some modifications. Briefly, 30‍ ‍μL of the GVv1 cell suspension adjusted to OD_600_=1 was inoculated into 10‍ ‍mL of LB broth supplemented with 0.5‍ ‍mg L-tryptophan and incubated at 30°C for 48‍ ‍h with shaking at 200‍ ‍rpm. After the incubation, the culture broth was centrifuged at 6,574×*g* for 10‍ ‍min. One milliliter of the resulting supernatant was then mixed with an equal volume of Salkowski reagent (1‍ ‍mL of 0.5 M FeCl_3_ in 50‍ ‍mL of 35% perchloric acid) (Gordon and Weber, 1951). The development of a red color, indicating IAA production, was measured at 530‍ ‍nm using a spectrophotometer (BioTek Epoch 2 Microplate Spectrophotometer; Agilent). The IAA content was quantified by comparisons to a standard curve generated with pure IAA. Results were expressed as the mean of three replicates.

#### Siderophore production assay

Siderophore production by GVv1 was assessed using the blue agar CAS assay, as described by Louden *et al.* (2011). Briefly, GVv1 was grown on TSA at 30°C for 24‍ ‍h and spot-inoculated at the center of CAS agar plates. After a 5-day incubation at 30°C, the plates were visually inspected for color changes. To avoid the contamination of iron traces, all glassware was washed with 1 M HCl and rinsed thrice with SDW. A positive result was indicated by a yellow or orange halo around the colonies. The experiment was repeated thrice.

#### Phosphate-solubilizing activity

To assess GVv1 phosphate solubilization ability, bacteria were grown on TSA at 30°C for 24‍ ‍h and spot-inoculated onto Pikovskaya agar medium (Pikovskaya, 1948). After the incubation of plates at 30°C for 5 days, phosphate solubilization was assessed by observing the formation of clear zones around the bacterial colonies, indicative of phosphate-solubilizing activity. Plates were prepared in triplicate.

#### Assessment of 1-aminocyclopropane-1-carboxylate (ACC) deaminase activity

ACC deaminase activity was measured following the protocol outlined by Dell’Amico *et al.* (2005). Briefly, a 50-μL aliquot of the GVv1 bacterial suspension adjusted to OD_600_=1 was inoculated into 3‍ ‍mL of Dworkin-Foster (DF) medium supplemented with 3.0‍ ‍mM ACC as the sole nitrogen source and incubated at 30°C for 96‍ ‍h with shaking at 200‍ ‍rpm. As a control, GVv1 was similarly cultured in DF medium without ACC. After the incubation, the cell density of GVv1 was assessed by measuring OD_600_. Results are the mean of three different replicates.

#### GVv1 identification by whole-genome sequencing

Genomic DNA (gDNA) was extracted from GVv1 as follows. GVv1 was cultured on LB broth at 30°C for 24‍ ‍h at 200‍ ‍rpm. Cells were harvested from the culture broth by centrifugation (1,600×*g*, 10‍ ‍min) and resuspended with 100‍ ‍mL of 1× TE buffer (to a total volume of 100‍ ‍mL; 1‍ ‍mL of 1 M Tris-HCl [pH 8.0], 200‍ ‍μL of 0.5‍ ‍M EDTA, and 98.8‍ ‍mL MilliQ). After another centrifugation at 1,600×*g* for 10‍ ‍min, the supernatant was removed, and the pellet was resuspended with 2‍ ‍mL of 1× TE buffer. A total of 150‍ ‍μL of 5‍ ‍M NaCl, 500‍ ‍μL of 0.5 M EDTA, 500‍ ‍μL lysozyme (10‍ ‍mg mL^–1^), and 50‍ ‍μL RNase (10‍ ‍mg mL^–1^) were added before an incubation at 37°C for 2 h. After the incubation, 125‍ ‍μL of 1 M Tris-HCl, 125‍ ‍μL of 10% SDS, and 7.5‍ ‍μL proteinase K (10‍ ‍mg mL^–1^) were added and incubated again at 60°C for 10‍ ‍min. One milliliter of 5 M NaCl and 800‍ ‍μL of 10% CTAB were added and incubated at 65°C for another 10‍ ‍min. An equivalent volume of 25:24:1 (v/v/v) phenol/chloroform/isoamyl alcohol (PCI) solution was then added to the mixture and centrifuged at 1,600×*g* for 10‍ ‍min. The aqueous layer containing DNA was carefully transferred to a new tube and mixed with an equal volume of 24:1 (v/v) chloroform/isoamyl alcohol. After centrifugation at 1,600×*g* for 10‍ ‍min, the upper layer was transferred to a new tube containing 3.2‍ ‍mL of 2-propanol and centrifuged again under the same conditions to precipitate gDNA. Thereafter, the sample was washed twice with 70% ethanol by centrifugation at 600×*g* for 5‍ ‍min and resuspended with 100‍ ‍μL of 1× TE buffer.

gDNA purification was performed as follows: 100‍ ‍μL of extracted gDNA was incubated at 37°C for 1‍ ‍h with 1‍ ‍μL RNase (10‍ ‍mg mL^–1^). An equivalent volume of PCI was added to the mixture and centrifuged at 6,400×*g* for 10‍ ‍min. After centrifugation, the resultant aqueous layer was transferred to a new tube containing 10‍ ‍μL of 3 M sodium acetate (pH 5.8) and 275‍ ‍μL of 99.5% ethanol and incubated at room temperature for 20‍ ‍min before centrifugation (8,000×*g*, 20‍ ‍min). The sample was subjected twice to a 70% ethanol wash by centrifugation at 8,000×*g* for 10‍ ‍min and resuspended into 50‍ ‍μL of 1× TE buffer. The integrity of extracted DNA was checked by 1.5% agarose gel electrophoresis, and DNA quality was assessed by measuring A260/A280 on a NanoDrop OneC ultraviolet-visible spectrophotometer (Thermo Fisher Scientific).

Before the genomic ana­lysis, short DNA fragments (<25‍ ‍kb) were depleted from the DNA sample, as described in the manufacturer’s instructions for the Short Read Eliminator (PacBio). The concentration of double-stranded DNA (dsDNA) was assessed using the sensitive fluorescence dye-based Qubit^TM^ 1× dsDNA HS Assay Kit (Invitrogen) on a Qubit^TM^ 4 fluorometer (Invitrogen). The DNA library was prepared using the Rapid Barcoding Kit 24‍ ‍V14 (SQK-RBK114.24; Oxford Nanopore Technologies). Sequencing was performed by loading samples onto the Flongle Flow Cell FLO-FLG114 on a MinION Mk1C platform (Oxford Nanopore Technologies) set at 230 Hz at 40°C for 24 h, with a quality score of 9 on Minknow version 24.06.8. Base calling was performed using Guppy version 6.5.7. Raw reads were trimmed with Filtlong version 0.2.1, and read lengths <1,000 bp were discarded. The draft genome was assembled using Trycycler version 0.5.4 (including the following packages: Flye 2.9.2-b1786, Minipolish version 0.1.2, and Raven 1.8enter.1) and polished with Racon 1.5.1. Genome annotation was performed with contigs obtained through the genome service DNA Data Bank of Japan (DDBJ) Fast Annotation and Submission Tool (Tanizawa *et al.*, 2016). The phylogenomic relationships of GVv1 and bacterial strains with high similarity according to average nucleotide identity (ANI) values were analyzed using the software package CheckM version 1.2.3 (Parks *et al.*, 2015).

### Statistical ana­lysis

All statistical ana­lyses were performed with EZR version 1.41, a graphical user interface for R (The R Foundation for Statistical Computing version 3.6.1).

### Nucleotide sequence accession numbers

The draft genome sequences of GVv1 were deposited in the DDBJ database under accession no. AP038752 and AP038753.

## Results

### Bacterial isolation from the legume rhizosphere

Seventy-three bacterial strains were isolated from the rhizosphere soil of seven legume species (Table S1). Three strains (strain codes: GGs1–GGs3) were from wild soybean (*Glycine soja*), 29 (GGm1–GGm29) from soybean (*G. max*), 5 (GVs1–GVs5) from common vetch (*Vigna sativa*), 5 (GVv1–GVv5) from hairy vetch (*Vicia villosa*), 8 (GAb1–GAb8) from hog-peanut (*Amphicarpaea bracteata* subsp. *edgeworthii*), 18 (TVa1–TVa18) from black adzuki bean (*Vigna angularis*), and 5 (GVh1–GVh5) from tiny vetch (*Vicia hirsuta*).

### Screening of the candidate biocontrol strain

#### Primary screening

In primary screening, the biocontrol efficacies of the isolated bacterial strains were evaluated on soybeans in two separate trials against PRSR. Seven days after the challenge inoculation with *P. sojae*, control plants exhibited root necrosis and stunted growth. In trial 1, 17 of 73 strains showed >50% control efficiency and were selected for trial 2. The results of trial 2 (Table S2) showed that the highest disease control was achieved by the GVv1, GGs2, GVs2, GGm21, TVa11, and GVv2 treatments, which ranged between 60.0 and 73.3% control efficiency. Based on these results, these six strains were selected for subsequent experiments.

#### Second screening under greenhouse conditions

The biocontrol efficacies of six strains (GVv1, GGs2, GVs2, GGm21, TVa11, and GVv2) selected in primary screening were further evaluated in two independent pot trials using the oospore inoculum under greenhouse conditions. Control plants showed browning of the taproot, reduced lateral root formation, and stunted growth (Fig. S1). The mean disease scores of control plants at 9 dpi reached 2.2 and 3.0 in trials 1 and 2, respectively. Plants treated with strain TVa11, GVv2, GGm21, Gvs2, or GGs2 showed similar or more severe symptoms than control plants in one or two trials (Table S3). In contrast, plants treated with GVv1 showed milder root browning than the other treatments in both trials (Fig. S1). The control efficiencies of GVv1 in trials 1 and 2 were 54.5 and 33.3%, respectively. Based on this result, GVv1 was selected as the final biocontrol candidate and exami­ned in subsequent experiments.

#### Identification of GVv1 by genome sequencing

Sequencing yielded 15,991 reads (mean lead length, 6,149 bp; N50 15,431 bp). The resulting draft genome of GVv1 had a total sequence length of 5,057,950 bp with a GC content of 55.4%, including 4,903 coding sequences, 25‍ ‍rRNA genes, 85 tRNA genes, and a coding ratio of 88.4%. Completeness was 99.54%, with 0.7% contamination. The ANI value between GVv1 with *Enterobacter pseudoroggenkampii* strain 155092T (accession no. GCA_026420145) was 98.32% (Table 2). These values were higher than the suggested threshold values for species delineation (95–96% for ANI). Therefore, GVv1 was identified as a new strain of *E. pseudoroggenkampii*.

#### Comparison of the disease control effect of GVv1 with the fungicide mancozeb-metalaxyl

The efficacy of GVv1 against PRSR was compared to that of the chemical fungicide mancozeb-metalaxyl in two independent pot experiments in the greenhouse (referred to as trials 1 and 2). At 9 dpi, control plants showed dark brown areas (necrotic symptoms) in the roots and reduced root development (Fig. S2). The mean disease scores of control plants were 1.6 and 2.1 in trials 1 and 2, respectively (Table 3). In contrast, seedlings pretreated with GVv1 or metalaxyl-mancozeb showed fewer necrotic symptoms in the roots and more lateral roots than control plants (Fig. S2). In trial 1, the GVv1 and metalaxyl-mancozeb treatments significantly reduced mean disease scores by 69.2 and 76.9%, respectively (Table 3). Similarly, in trial 2, the GVv1 and mancozeb-metalaxyl treatments significantly reduced mean disease scores from that of the control, with control efficiencies of 70.6 and 88.2%, respectively. Between the GVv1 and fungicide treatments, there was no significant difference in any of the trials.

#### Inhibitory activity of GVv1 against *P. sojae* mycelial growth

**Fig. 1.**
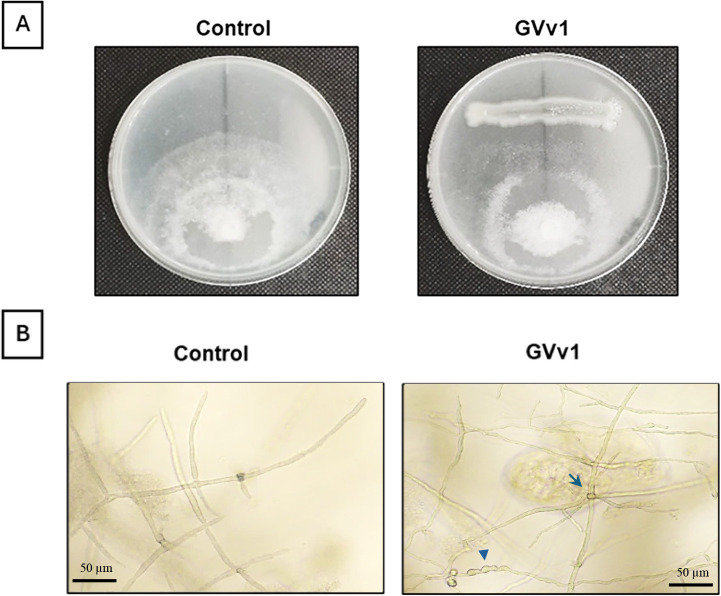
Inhibition of *Phytophthora sojae* hyphal growth by GVv1 on PDA. (A) Photographs of representative plates taken at 12 dpi. (B) Photomicrographs of *P. sojae* hyphae in the confrontation zone. The arrowhead shows hyphal swelling, and the arrow points to excessive branching.

The inhibitory activities of GVv1 and three less or non-protective strains against the hyphal growth of *P. sojae* were evaluated using the dual-culture method. GVv1 inhibited the hyphal growth of the pathogen, resulting in a significant reduction in the radius of the fungal colony (Fig. 1A and Table 4). The inhibitory activities of the less or non-protective strains varied among the strains. One of the less protective strains, GVh3, exhibited similar inhibitory activity to that of GVv1, while another less protective strain, GVh5, showed no activity. Conversely, the non-protective strain GVs5 exhibited the highest inhibitory activity. Fig. 1B shows the hyphal morphology of *P. sojae* at the colony periphery facing the GVv1 colony. In comparison to hyphae in the control plate, some hyphae in the confrontation zone close to GVv1 were swollen and showed unusual branching.

#### Effects of daidzein and soybean root exudates on GVv1 growth

**Fig. 2.**
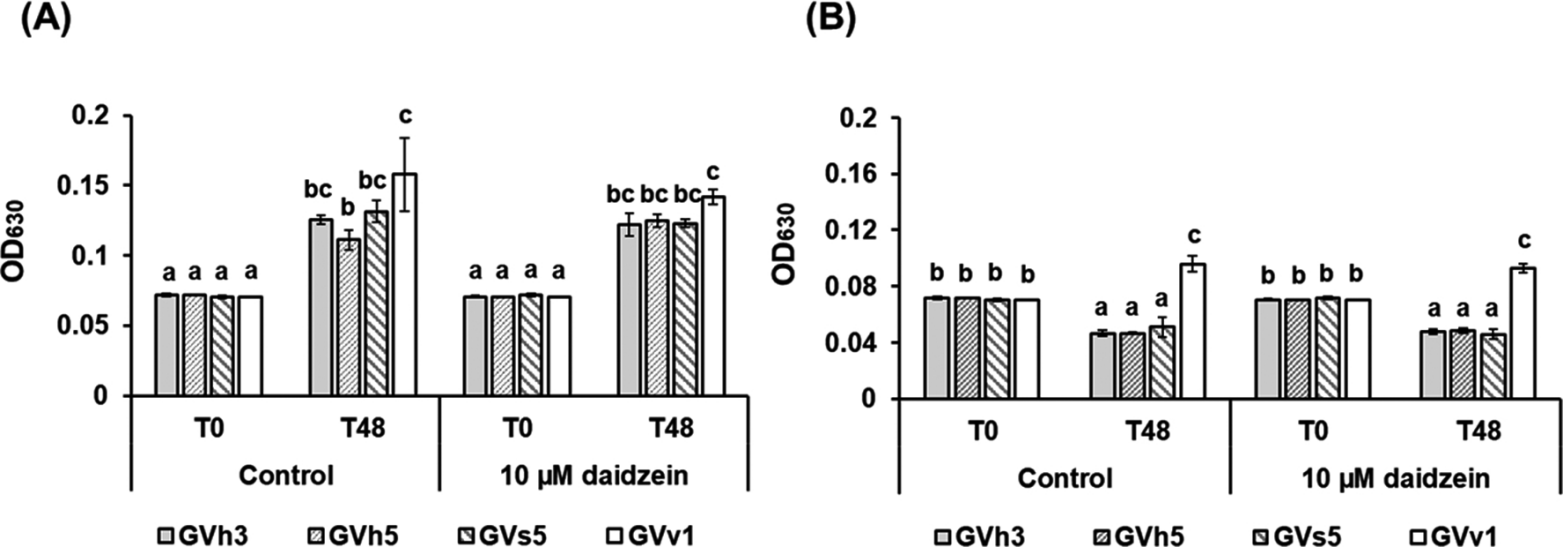
Relative growth of GVv1 and three other strains with no or weak (<15%) control efficiency in primary screening against PRSR (GVh3, GVh5 and GVs5) in 10-fold LB broth (A) or soybean root exudates (B). Bars represent OD_630_ before (T0) and after a 48-h incubation (T48) with or without the external application of daidzein to a final concentration of 10‍ ‍μM. Bars show the mean±SD of three individual experiments. The significance of differences is denoted by different letters based on Tukey’s HSD test (*P*<0.05).

Three less or non-protective strains (GVh3, GVh5, and GVs5) showed similar proliferation in 10‍ ‍μM daidzein-supplemented and unsupplemented 1/10 LB media (Fig. 2A). GVv1 also proliferated similarly in both daidzein-supplemented and unsupplemented media. Cell densities in both media after the 48-h culture of GVv1 were higher than those of the less or non-protective strains.

In soybean root exudates, the population of the less or non-protective strains significantly decreased regardless of daidzein supplementation (Fig. 2B). In contrast, the population of GVv1 significantly increased in both daidzein-supplemented and unsupplemented root exudates.

#### GVv1 hemolytic activity

GVv1 did not change the color of blood agar (Fig. S3), indicating that it does not exhibit hemolytic activity.

#### Plant growth-promoting activity of GVv1 on soybean

**Fig. 3.**
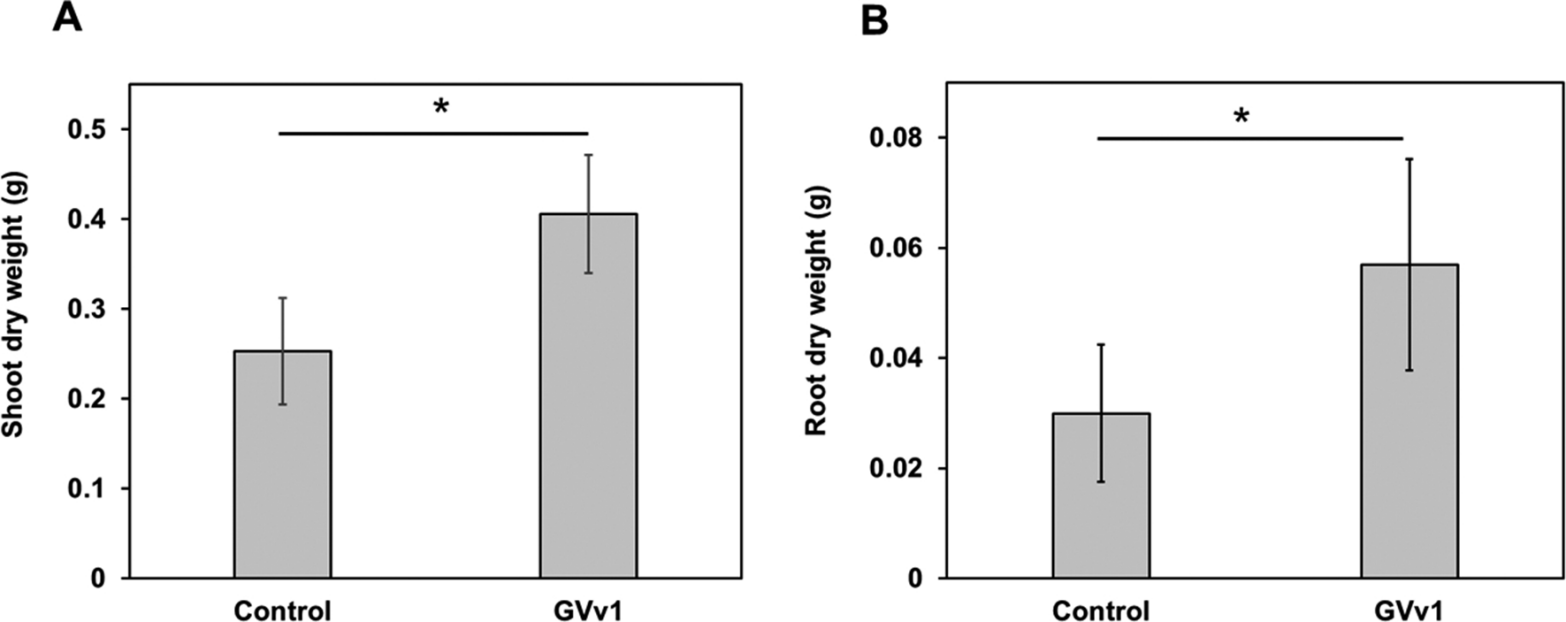
Effect of GVv1 drench application on soybean growth at 24 dpi under greenhouse conditions. The means of shoot dry weight (A) and root dry weight (B) from three independent trials are shown. Error bars show means±SD. Significant differences between the control and GVv1 treatments are denoted by asterisks, as assessed by the Student’s *t*-test (*P*<0.05).

The drenching application of GVv1 to soybean seeds resulted in more shoot growth and root development than in control plants 24 days after inoculation. GVv1-treated plants showed a 64.0% increase in shoot dry weight and a 100% increase in root dry weight from those of control plants. All parameters measured showed significant differences (the Student’s *t*-test, *P*<0.05) between GVv1-treated and control plants (Fig. 3).

#### Characteristics of GVv1 related to plant growth-promoting activity

The characteristics of GVv1 related to plant growth-promoting activity were exami­ned *in vitro*. The results obtained showed that this strain produced large amounts of IAA (86.03‍ ‍μg mL^–1^) during the 48-h incubation on LB medium supplemented with L-tryptophan (Table 5). The development of a yellow halo on CAS agar and a clear zone on Pikovskaya agar around the colony indicated that GVv1 produced siderophores and solubilized phosphate, respectively (Fig. S4). Furthermore, GVv1 proliferated on DF minimal medium supplemented with ACC, but not on DF medium without ACC, indicating that GVv1 exhibited ACC deaminase activity (Fig. S5).

## Discussion

The present study aimed to identify a BCA that controls the PRSR of soybean caused by *P. sojae*. To achieve this, 73 rhizobacteria were isolated from wild and cultivated legumes and screened for their biocontrol activity against PRSR in pot experiments. GVv1 isolated from hairy vetch (*V. villosa*) exerted the strongest and most consistent protective effect among the strains tested. The control efficacy of GVv1 was similar to that of the commercial fungicide mancozeb-metalaxyl.

GVv1 suppressed disease severity induced by the zoospore inoculation (Table S2 and 3) and oospore inoculation (Table S3). Under natural conditions, *P. sojae* remains dormant as oospores in soil and plant debris. When soil moisture increases, oospores germinate to form mycelia and eventually sporangia, which germinate directly to form new hyphae or produce zoospores that infect soybean roots (Dorrance *et al.*, 2007). BCAs effective against multiple stages of the oomycete life cycle have better biocontrol performance than those that only affect one stage (Paulitz *et al.*, 1992; Zohara *et al.*, 2016). Therefore, GVv1 is expected to be effective against PRSR under field conditions.

The taxonogenomic ana­lysis indicated that the GVv1 genome showed the highest ANI (98.32%) to that of the‍ ‍genome of *E. pseudoroggenkampii* strain 155092 (GCA_026420145). This value was above the threshold for species delineation (ANI cut-off >95%), indicating that GVv1 is *E. pseudoroggenkampii*. *Enterobacter* species are common rhizosphere colonizers of many plants, including soybean, with some species enhancing the abiotic stress tolerance or growth of host plants (Roberts *et al.*, 1992; Rattray *et al.*, 1995; Ramesh *et al.*, 2014). Many *Enterobacter* strains possess properties related to plant growth-promoting activity, such as nitrogen fixation, soil phosphorus solubilization, and 1-aminocyclopropane-1-carboxylate deaminase activity. Additionally, they have been shown to possess properties related to biocontrol activity against plant pathogens, such as the production of lytic enzymes, antibiotics, and siderophores, making them promising microbial inoculants for improving crop productivity (Jha *et al.*, 2011). Several *Enterobacter* strains isolated from plants exhibit biocontrol activity against various plant diseases, including those caused by oomycetes (Okamoto *et al.*, 2000; Al-Mughrabi, 2010; Kazerooni *et al.*, 2020). However, to the best of our knowledge, the biocontrol potential of *Enterobacter* species against PRSR on soybean plants has yet to be reported. *E. pseudoroggenkampii* is a new species recently identified from the pus of the diabetic foot ulcers of a patient, as reported by Wu *et al.* (2023). Due to its origin, this species may be at risk of becoming an opportunistic human pathogen. Wu *et al.* (2023) detected virulence genes,‍ ‍such as aerobactin-encoding iucABCD-iutA and salmochelin-encoding iroN, from its genome. However, there is no evidence to clarify the involvement of this species in human pathogenicity; therefore, its clinical relevance remains unclear. Importantly, GVv1 did not exhibit hemolytic activity, a trait commonly associated with pathogenicity in animals and humans (Thakkar *et al.*, 2015), suggesting that it does not pose a risk to humans or animals. However, further risk assessments are needed to fully confirm the safety of this strain.

Various mechanisms, such as competition for space and nutrients, the enhancement of host resistance, and antibiosis, play a role in the biocontrol of plant diseases (Köhl *et al.*, 2019). Antibiosis is one of the most commonly described mechanisms and has been reported in many *Enterobacter* species against phytopathogenic *Phytophthora* species (Okamoto *et al.*, 2000; Toh *et al.*, 2016; Admassie *et al.*, 2023). For example, *Enterobacter aerogenes* strain B8 isolated from soil has been shown to cause extensive cell membrane disruption, organelle aggregation, necrosis, vacuolation, and cell wall breakdown in *Phytophthora cactorum*, ultimately leading to hyphal death (Brewster *et al.*, 1997). In the dual-culture assay, GVv1 caused the swelling and abnormal branching of *P. sojae* hyphae, resulting in hyphal growth inhibition (Fig. 1B). These abnormalities on oomycetes were induced via the secretion of certain metabolites, such as 2,4-diacetylphloroglucinol (DAPG) produced by *Pseudomonas fluorescens*, or cyclopeptides, including iturin A and massetolide A produced by *Bacillus subtilis* and *P. fluorescens*, respectively (van de Mortel *et al.*, 2009; Islam and Fukushi, 2010) (Wang *et al.*,‍ ‍2019 Inhibitory effect of *Bacillus subtilis* WL-2 and its‍ ‍IturinA lipopeptides against *Phytophthora infestans*. bioRxiv 751131). Some *Enterobacter* strains also produce iturins and DAPG (Picard *et al.*, 2000; Mandal *et al.*, 2013). The oomycete cell wall is largely composed of β-1,3-glucan, β-1,6-glucan, cellulose, and small amounts of chitin; therefore, it may be a target for hydrolytic enzymes, such as cellulase, chitinase, and endoglucanase (Sietsma *et al.*, 1969; Schlumbaum *et al.*, 1986; De la Cruz-Quiroz *et al.*, 2018; Cheng *et al.*, 2019). These enzymes have been identified in *Enterobacter roggenkampii*, a strain closely related to GVv1 phylogenetically and may be involved in the hyphal inhibition observed (Guo *et al.*, 2020). In the present study, we found that even strains that did not efficiently control PRSR on soybean plants exhibited equal or stronger inhibitory activity against *P. sojae* hyphal growth (Table 4). Therefore, the production of antifungal metabolites and/or hydrolytic enzymes may contribute to, but does not fully explain, the mechanism of PRSR suppression by GVv1.

Effective root colonization is essential for BCAs to exert biocontrol activity against soil-borne pathogens (Whipps, 2001). One of the factors that affects the persistence of BCAs in the rhizosphere is their tolerance to antimicrobial compounds released by host plant roots (el Zahar Haichar *et al.*, 2014). Daidzein is a major isoflavone secreted by soybean roots into the rhizosphere and may act as a repellent to assemble bacterial communities in the rhizosphere (Guo *et al.*, 2011; Wang *et al.*, 2012; Okutani *et al.*, 2020). Aoki *et al.* (2024) showed that daidzein-non-degrading bacterial strains were susceptible to daidzein at a concentration of 10‍ ‍μM, which is within the range reported to be secreted into the soybean rhizosphere (Graham, 1991). We hypothesized that daidzein-tolerant or daidzein-utilizing bacteria may be good candidates for the biocontrol of PRSR. We found that GVv1 was tolerant to daidzein and grew in the medium containing 10‍ ‍μM daidzein (Fig. 2). Moreover, the growth rate of GVv1 was higher than that of the bacterial strains with low or no biocontrol efficiency. The tolerance of GVv1 to the naturally occurring concentration of daidzein and the higher growth rate of this strain may support the rapid and stable colonization of GVv1 in the soybean rhizosphere. However, less or non-protective strains were also tolerant to the same concentration of daidzein, suggested that daidzein tolerance is not be the key characteristic explaining the superior biocontrol effect of GVv1. Notably, GVv1 proliferated in soybean root exudates, while the population of less or non-protective strains decreased after the incubation. This result suggests that soybean root exudates contain antibacterial compounds other than daidzein, and GVv1 may be tolerant to these compounds. Soybean plants are known to secrete other antimicrobial compounds, such as genistein, glycitein, soyasaponins, and glyceollins, into the rhizosphere (Tsuno *et al.*, 2018; Sugiyama, 2019). These metabolites are also effective against various bacteria (Hong *et al.*, 2006; Bamji and Corbitt, 2017; Horie *et al.*, 2018; Lalouckova *et al.*, 2021). In the present study, we did not test the growth of GVv1 in a medium supplemented with these metabolites. However, the strain may also be tolerant to these compounds, potentially explaining its stronger and more consistent biocontrol effect against PRSR than other strains. Further detailed studies are needed to validate this hypothesis.

As described above, many *Enterobacter* strains promote the growth of various crops, including soybeans (Kuklinsky-Sobral *et al.*, 2004; Ramesh *et al.*, 2014). Consistent with these findings, GVv1 exhibited strong plant growth-promoting activity in soybean plants (Fig. 3). One of the most reported and significant characteristics associated with the plant growth-promoting effect of rhizobacteria is the production of phytohormones, particularly auxins, such as IAA (Ahmed and Hasnain, 2014). Endogenous IAA is involved in various physiological development processes, such as cell division and elongation, tissue differentiation, and lateral root formation (Fu *et al.*, 2015). An *in vitro* assessment of IAA production showed that GVv1 produced large amounts (86.03‍ ‍g mL^–1^) in LB medium supplemented with L-tryptophan, which is higher than that by most IAA-producing plant growth-promoting rhizobacteria (PGPR), typically ranging between ~5 and 70‍ ‍mg L^–1^ (Mohite, 2013; Herlina *et al.*, 2017). The production of large amounts of IAA by PGPR has been associated with a greater increase in plant weight (Mohite, 2013). The facilitation of nutrient uptake by rhizobacteria is another mechanism that is crucial for enhancing plant growth. Essential nutrients, such as phosphorus and iron, are normally present in non-soluble forms that cannot be assimilated by plants (Pattnaik *et al.*, 2021). GVv1 may solubilize phosphate and secrete siderophores (Fig. S4), which may enhance phosphorus and iron uptake and increase soybean growth. In addition, GVv1 exhibited ACC deaminase activity (Table 5). ACC deaminase regulates plant levels of ethylene, a hormone whose accumulation may be detrimental under stress conditions (Moon and Ali, 2022). ACC deaminase activity reduces the impact of several abiotic stresses, such as flooding, drought, salt, metals, or organic contaminants, and even improves nodulation in legumes under normal conditions, translating into a plant growth-promoting effect that may explain the soybean growth increase by GVv1 (Glick, 2014).

In conclusion, the present study identified *E. pseudoroggenkampii* GVv1 as a promising BCA that suppresses PRSR and stimulates soybean growth. Its application may contribute to the development of novel biocontrol products that improve soybean production and sustainability. Further research is needed to assess the safety and field performance of GVv1 in order to ensure its suitability for practical agricultural use.

## Citation

Taboadela-Hernanz, J., Ikagawa, Y., Yamauchi, K., Minoshima, Y., Suga, H., and Shimizu, M. (2025) Biocontrol of *Phytophthora* Root and Stem Rot and Growth Promotion of Soybean Plants by the Rhizobacterium *Enterobacter pseudoroggenkampii* Strain GVv1 Isolated from *Vicia villosa* Roth. *Microbes Environ ***40**: ME24089.

https://doi.org/10.1264/jsme2.ME24089

## Supplementary Material

Supplementary Material

## Figures and Tables

**Table 1.** Legume samples used in this study to isolate rhizosphere bacteria.

Common name (scientific name)	Sampling site	Coordinates	Sampling date
Soybean (*Glycine max*)	Yanagido, Gifu city, Gifu Prefecture	35°28′05.1″N, 136°44′19.3″E	April 18, 2022
Common vetch (*Vigna sativa*)	Yanagido, Gifu city, Gifu Prefecture	35°27′43.2″N, 136°44′25.1″E	May 4, 2023
Hairy vetch (*Vicia villosa*)	Yanagido, Gifu city, Gifu Prefecture	35°27′43.4″N, 136°44′21.5″E	May 4, 2023
Tiny vetch (*Vicia hirsuta*)	Yanagido, Gifu city, Gifu Prefecture	35°27′43.3″N, 136°44′27.1″E	May 4, 2023
Wild soybean (*Glycine soja*)	Kuronominami, Gifu city, Gifu Prefecture	35°27′21.4″N, 136°43′30.0″E	June 20, 2022
Hog-peanut (*Amphicarpaea bracteata* subsp. *edgeworthii*)	Sano, Gifu city, Gifu Prefecture	35°31′04.7″N, 136°43′22.8″E	June 20, 2022
Black adzuki bean (*Vigna angularis*)	Kofu-cho, Tottori Prefecture	35°18′21.7″N, 133°30′05.9″E	September 28, 2022

**Table 2.** ANI values for GVv1 relative to type strains of *Enterobacter* with the highest ANI value (<90%).

Organism name	Type strain	Accession	ANI (%)	Align % (reference)	Align % (query)
*Enterobacter pseudoroggenkampii*	155092	GCA_ 026420145 . 1	98.32	91.67	88.18
*Enterobacter quasiroggenkampii*	WCHECL1060	GCA_ 001039365 . 2	94.85	84.84	80.62
*Enterobacter dykesii*	E1	GCA_ 008364625 . 1	94.55	86.7	77.65
*Enterobacter asburiae*	FDAARGOS_892	GCA_ 016027695 . 1	94.43	80.22	75.61
*Enterobacter vonholyi*	E13	GCA_ 008364555 . 1	94.31	85.28	77.4
*Enterobacter asburiae*	ATCC 35953	GCA_ 001521715 . 1	94.31	79.59	75.63
*Enterobacter roggenkampii*	DSM 16690	GCA_ 024390995 . 1	94.12	77.47	74.34
*Enterobacter roggenkampii*	FDAARGOS 1430	GCA_ 019047025 . 1	94.06	80.49	78.25
*Enterobacter roggenkampii*	DSM 16690	GCA_ 001729805 . 1	94.05	80.99	78.46
*Enterobacter quasimori*	90044	GCA_ 003964905 . 1	91.72	68.96	64.19

**Table 3.** Control efficiencies of GVv1 and mancozeb-metalaxyl fungicide against PRSR on soybean seedlings under greenhouse conditions.

Treatment	Trial 1			Trial 2	
	Disease score*	Control efficiency		Disease score	Control efficiency
Control	1.6±1.2 a			2.1±0.6 a	
GVv1	0.5±0.5 b	69.2		0.6±1.1 b	70.6
Mancozeb-metalaxyl	0.4±0.5 b	76.9		0.3±0.5 b	88.2

* Each value represents the mean±standard deviation of five replicates. Different letters indicate significant differences between treatments (the Mann–Whitney U test, *P*<0.05).

**Table 4.** Inhibitory activities of GVv1 and less or non-protective strains against the hyphal growth of *Phytophthora sojae*.

Treatment	Radius of *P. sojae* colony (cm)
Control	5.9±0.08 a
GVv1	5.2±0.05 b
GVh3	5.3±0.05 b
GVh5	5.9±0.05 a
GVs5	1.9±0.12 c

Each value represents the mean±standard deviation of three replicates. Different letters indicate significant differences between treatments (Tukey’s HSD, *P*<0.05).

**Table 5.** *In vitro* traits of GVv1 related to plant growth-promoting activity.

Strain	IAA production (μg mL^–1^)	Siderophore production	Phosphate solubilization	ACC deaminase activity
GVv1	86.03±2.8	+	+	+

The value represents the mean±standard deviation of three replicates. “+”: positive.
