# Supplementary material for: Biocontrol of Phytophthora Root and Stem Rot and Growth Promotion of Soybean Plants by the Rhizobacterium Enterobacter pseudoroggenkampii Strain GVv1 Isolated from Vicia villosa Roth
Source: Microbes Environ. 2025 May 1;40(2):ME24089. doi: 10.1264/jsme2.ME24089 (PMC12213067; doi:10.1264/jsme2.ME24089)
Supplement: Supplementary file 1 — Supplementary Material [file 40_24089_s1.pdf]

Table S1. Summary of bacterial isolates obtained from the rhizosphere of legume plants employed in this study. The table lists each strain's name and the specific legume host plant from which it was obtained.

**Strain**	**Plant source**
GGs1–GGs3	Wild soybean
GGm1–GGm29	Soybean
GVs1–GVs5	Common vetch
GVv1–GVv5	Hairy vetch
GAb1–GAb8	Hog peanut
TVa1–TVa18	Black adzuki bean
GVh1–GVh5	Tiny vetch

Table S2. Control efficiency of bacterial strains against PRSR on soybean seedlings in trial 2 of the primary screening under the controlled conditions.

Treatment	Disease score*	Control efficiency
Control	3.8 ± 0.4	
GAb3	2.8 ± 1.6	26.7
GAb7	4 ± 0.0	–6.7
GGm1	4 ± 0.0	–6.7
GGm15	3 ± 1.7	20.0
GGm21	1.3 ± 1.1	66.7
GGm6	3 ± 1.2	20.0
GGs2	1.5 ± 1.5	60.0
GVh3	3.3 ± 1.3	13.3
GVh5	3.8 ± 0.8	0.0
GVs2	1.3 ± 1.3	66.7
GVs3	3 ± 1.0	20.0
GVs5	3.3 ± 0.8	13.3
GVv1	1.5 ± 1.5	60.0
GVv2	1 ± 0.7	73.3
TVa11	1.3 ± 1.1	66.7
TVa13	3.3 ± 1.3	13.3
TVa18	3 ± 1.2	20.0

*Data are the mean ± standard deviation (SD) of four replicates.

Table S3. Suppressive effect of six rhizobacterial strains against PRSR of soybean plants in the second screening experiment under greenhouse conditions.

Treatment	Trial 1		Trial 2	
	Disease score*	Control efficiency	Disease score	Control efficiency
Control	2.2 ± 1.3		3.0 ± 1.4	
GGm21	2.8 ± 1.3	–27.3	2.8 ± 1.6	6.7
GGs2	1.8 ± 1.5	18.2	3.4 ± 1.3	–13.3
GVs2	2.6 ± 1.3	–18.2	4.0 ± 0.0	–33.3
GVv1	1.0 ± 0.7	54.5	2.0 ± 1.2	33.3
GVv2	2.6 ± 0.6	–18.2	3.6 ± 0.6	–20.0
TVa11	2.8 ± 1.3	–27.3	2.4 ± 1.5	20.0

*Data are the mean ± SD of five replicates.

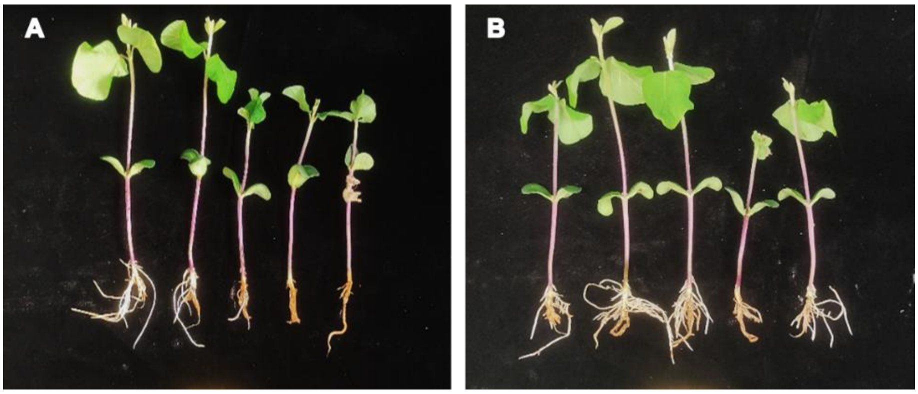

Fig. S1. PRSR Symptom development in control and GVv1-treated soybean seedlings. (A) Control plants show severe root necrosis and a reduced number of lateral roots. (B) GVv1-treated plants show milder root necrosis and improved lateral root growth than control plants. Photographs were taken 9 dpi (trial 2).

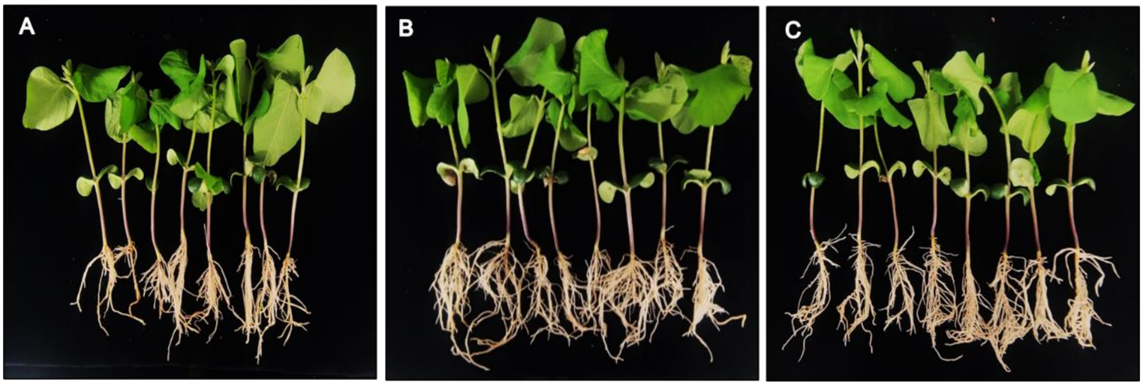

Fig. S2. Differences in PRSR symptoms in soybean seedlings among (A) control, (B) GVv1-treated, and (C) mancozeb-metalaxyl-treated soybean plants. Control plants had fewer lateral roots, and the remaining roots showed more brownish symptoms compared to fungicide or GVv1 treated plants. Photographs were taken 9 days after inoculation.

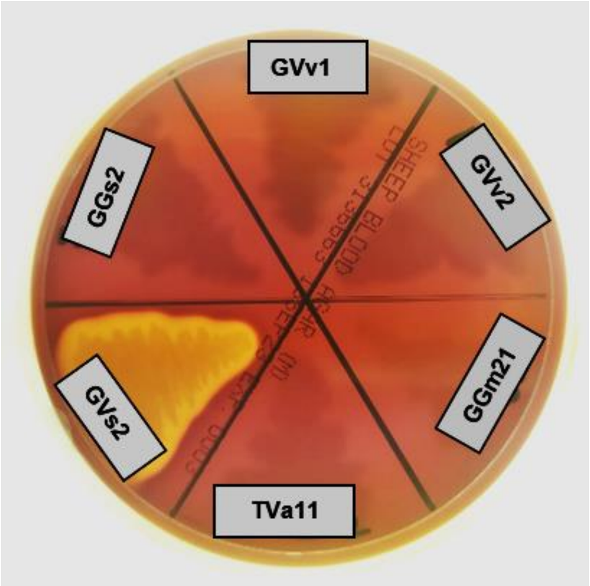

Fig. S3. Hemolytic activity on 5% sheep blood agar plate of six bacterial strains selected in the first screening. Strain GVs2 showed α-hemolysis as indicated by the yellow halo formation around the colony, but the remaining strains, including GVv1, did not show such changes. Photographs were taken at 48 h after incubation at 37°C.

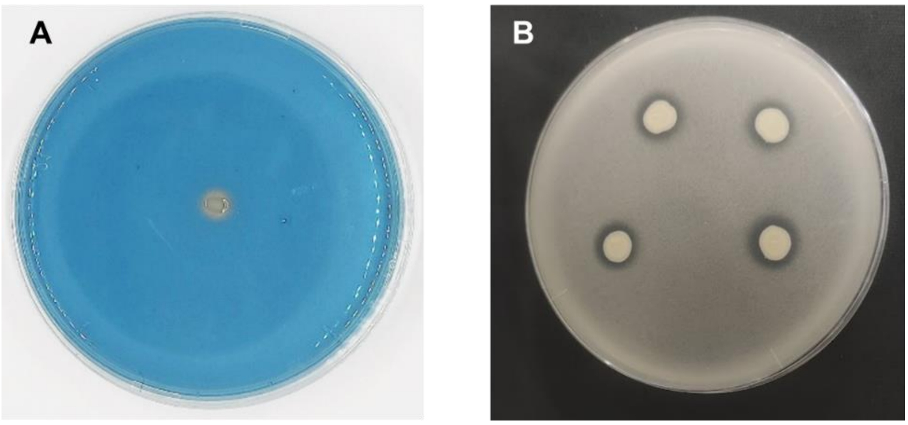

Fig. S4. Siderophore-producing and phosphate-solubilizing activities of GVv1. (A) GVv1 produced a yellow halo around the colony on blue CAS agar, indicating siderophore production. (B) GVv1 produced a clear zone around the colonies on Pikovskaya agar, indicating calcium phosphate solubilization. Photographs were taken 5 days after incubation at 30°C.

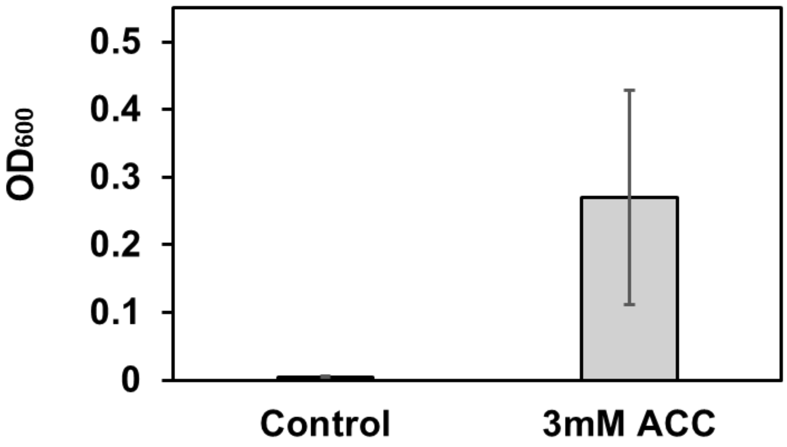

Fig. S5. Growth of GVv1 in DF medium supplemented with 3 mM ACC. Values of OD_{600} of GVv1 after incubation for 96 h in DF minimal medium without and with 3 mM ACC are shown. Error bars show ± standard deviation (SD) of the mean.
